# Effects of the Temperature and *Limosilactobacillus fermentum* Co-Inoculation on the Expression of AFB1-Synthesis Genes and the Level of Toxin Produced by *Aspergillus flavus* Zt41 in Corn Silage

**DOI:** 10.3390/toxins18060254

**Published:** 2026-06-04

**Authors:** Szilamér Ferenczi, Ildikó Bata-Vidács, Judit Kosztik, István Nagy, Katalin Inotai, Olívia Csernus, Natália Szeőcs, Zsuzsanna Szőke, Mónika Varga, András Szekeres, József Kukolya

**Affiliations:** 1 Laboratory of Molecular Neuroendocrinology, Institute of Experimental Medicine, Hungarian Research Network, H-1083 Budapest, Hungary; ferenczi.szilamer@koki.hun-ren.hu; 2 Department of Microbiology and Applied Biotechnology, Institute of Genetics and Biotechnology, Hungarian University of Agriculture and Life Sciences, H-2100 Gödöllő, Hungary; natalia.sz@cebiosys.com; 3 Centre of Research and Development, Eszterházy Károly Catholic University, H-3300 Eger, Hungary; kosztik.judit@uni-eszterhazy.hu (J.K.); istvan.nagy@uni-eszterhazy.hu (I.N.); kukolya.jozsef@uni-eszterhazy.hu (J.K.); 4 Agro-Environmental Research Centre, Institute of Environmental Sciences, Hungarian University of Agriculture and Life Sciences, H-2100 Gödöllő, Hungary; inotai.katalin@uni-mate.hu; 5 Section of Competent Authority for Biocides, Department for Chemical Safety and Competent Authorities, National Centre for Public Health and Pharmacy, H-1097 Budapest, Hungary; csernus.olivia@nngyk.gov.hu; 6 Central European Biosystems Ltd., H-1044 Budapest, Hungary; 7 Department of Animal Biotechnology, Institute of Genetics and Biotechnology, Hungarian University of Agriculture and Life Sciences, H-2100 Gödöllő, Hungary; 8 Department of Microbiology, Faculty of Science and Informatics, University of Szeged, H-6726 Szeged, Hungary; varga.j.monika@gmail.com (M.V.); szandras@bio.u-szeged.hu (A.S.)

**Keywords:** aflatoxin B1, gene expression, *aflR*, *cypA*, *ordA*, *omtA*, *ver1*, *Limosilactobacillus fermentum*

## Abstract

Aflatoxin B1 (AFB1), a highly potent Group 1 human carcinogen produced by *Aspergillus flavus* (*A. flavus*), poses a significant contamination risk to corn silage, a threat that is further intensified by rising global temperatures. This study aimed to characterize the combined effects of temperature and co-inoculation with the lactic acid bacterium *Limosilactobacillus fermentum* (*L. fermentum*) on AFB1 production and the expression of key biosynthetic genes in *A. flavus* colonizing corn silage. Corn silage was incubated at 20 °C, 30 °C, and 37 °C with and without *L. fermentum*. Using qRT-PCR and HPLC, we found that elevated temperatures, particularly 37 °C, strongly induced the expression of the aflatoxin biosynthetic cluster, including the regulatory gene *aflR* and structural genes such as *omtA* and *ordA*. Co-inoculation with *L. fermentum* consistently reduced in the final AFB1 concentration by approximately 50–60% at all three temperatures. Molecular analysis revealed that this reduction was associated with transcriptional repression at 30 °C and 37 °C. *L. fermentum* consistently and markedly down-regulated the expression of *aflR* and all structural genes. A particularly pronounced suppression was observed for the late-pathway gene *ordA* at 30 °C. These findings provide molecular evidence supporting the incorporation of selected *L. fermentum* strains into silage inoculant formulations to mitigate the AFB1 risk under high-temperature conditions.

## 1. Introduction

Aflatoxin B1 (AFB1), produced primarily by *A. flavus* and *A. parasiticus*, is the most potent naturally occurring hepatocarcinogen and is classified as a Group 1 human carcinogen by the International Agency for Research on Cancer [1,2]. Contamination of maize and maize-derived products, including silage, poses serious risks to both human and animal health and contributes to economic losses worldwide [3,4,5,6]. Global climate change exacerbates this problem by creating warmer and more humid conditions that favor aflatoxigenic *Aspergillus* spp. and AFB1 production [7,8,9]. The aflatoxin biosynthetic pathway in *A. flavus* comprises more than 30 clustered genes spanning approximately 80 kb [10]. The pathway-specific transcriptional regulator AflR, encoded by *aflR*, is essential for activating most structural genes [11,12]. In the present study, we investigated four key late-pathway structural genes: *omtA* (*aflP*), encoding an O-methyltransferase; *cypA* (*aflU*), encoding a cytochrome P450 monooxygenase; *ver1* (*aflM)*, involved in the conversion of versicolorin A; and *ordA* (*aflQ*), encoding the cytochrome P450 monooxygenase that catalyzes the final conversion of O-methylsterigmatocystin to AFB1.

The expression of *aflR* and downstream structural genes is highly sensitive to environmental cues, particularly temperature, water activity (aw), pH, oxidative stress, and carbon dioxide levels [13,14,15,16,17]. Recent studies using RNA-Seq and qPCR have shown that temperature and water stress can significantly modulate the expression of *aflR*, *aflS*, and several structural genes, resulting in corresponding changes in AFB1 accumulation [18,19,20,21,22].

Among the numerous strategies proposed to mitigate aflatoxin contamination, biological control using lactic acid bacteria (LAB) has gained considerable attention because of its safety, natural origin, and compatibility with fermented foods and feeds [23,24]. Several *Lactobacillus* species, including *L. plantarum*, *L. pentosus*, *L. fermentum*, and *L. casei*, have been shown to inhibit *A. flavus* growth and/or AFB1 production in vitro and in various food and feed systems [25]. These mechanisms may involve the inhibition of hyphal growth and spore formation, as well as the production of organic acids and, as recently demonstrated, the direct downregulation of aflatoxin biosynthetic genes [26]. However, many of the currently available physical, chemical, and biological mitigation methods still suffer from limitations such as incomplete efficacy under field conditions, potential impacts on feed quality, or high implementation costs, underscoring the need for more effective, sustainable, and environmentally compatible biocontrol approaches.

Despite these advances, few studies have systematically examined the combined effects of temperature and LAB co-inoculation on the expression of both regulatory (*aflR*) and lesser-studied structural genes (*cypA*/*aflU*, *ordA*/*aflQ*, *omtA*/*aflP*, and *ver1*/*aflM*) in a silage matrix, which represents a realistic post-harvest scenario for aflatoxin production.

Therefore, the present study aimed to (i) characterize the temperature-dependent expression patterns of key AFB1 biosynthetic genes in *A. flavus* Zt41 colonizing corn silage and (ii) evaluate the ability of *L. fermentum* to suppress gene expression and AFB1 accumulation under different thermal conditions.

## 2. Results

Gene expression and toxin analyses were performed on samples collected after the initial 7-day incubation with *A. flavus* (day 7 baseline, where applicable) and subsequently on days 12, 16, and 21. *L. fermentum* was added after day 7 to a subset of bottles. Its effects on gene expression were evaluated at 30 °C and 37 °C, while aflatoxin B1 (AFB1) concentrations were determined at the end of the experiment (day 21) at all three temperatures (20 °C, 30 °C, and 37 °C).

### 2.1. mRNA Expression Analysis of A. flavus Zt41 Genes Responsible for Toxin Production at 20 °C

**Figure 1:**
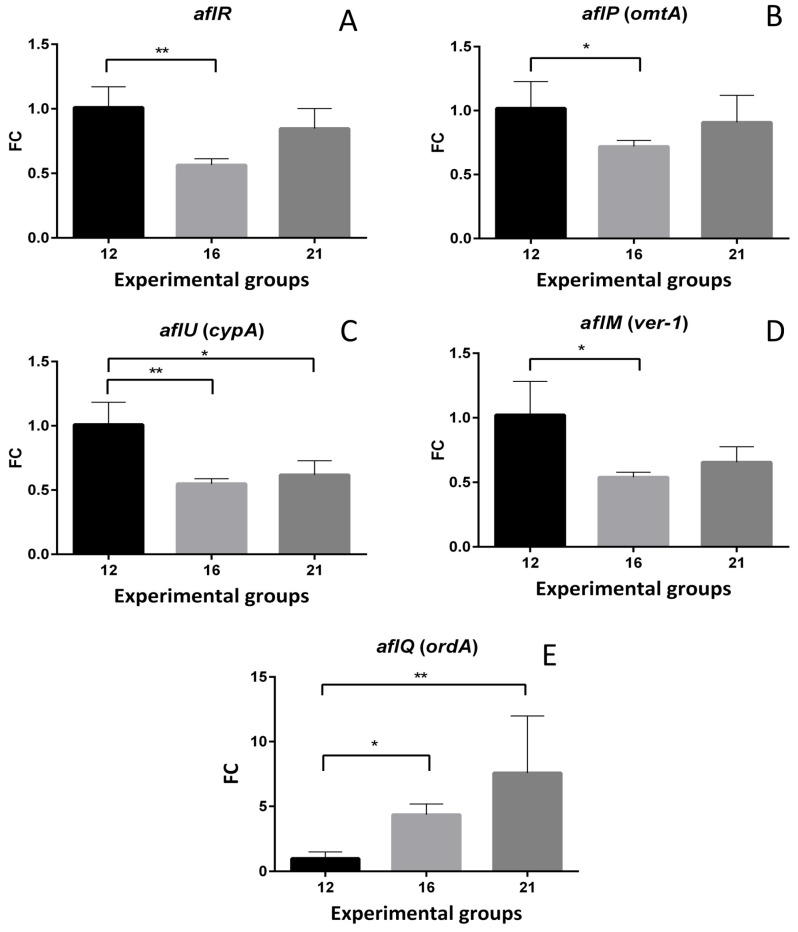
mRNA expression of *aflR* (**A**)*, aflP* (*omtA*) (**B**), *aflU* (*cypA*) (**C**), *aflM* (*ver-1*) (**D**), and *aflQ* (*ordA*) (**E**) genes from *A. flavus* samples incubated at 20 °C on days 12, 16, and 21. FC—Fold Change. The sampling days were 12, 16, and 21. The deviations were plotted with SD (Standard Deviation) values. The significances were *p* < 0.05 * and *p* < 0.01 **. Statistical comparisons were performed using one-way ANOVA with Bonferroni post hoc test. Asterisks indicate significant differences versus day 12 (*p* < 0.05 *, *p* < 0.01 **). Comparisons between day 16 and day 21 are indicated where significant. Non-significant differences are not marked for clarity.

At 20 °C (without *L. fermentum*), the expression of the regulatory gene *aflR* and the structural genes *omtA* (*aflP*), *cypA* (*aflU*), and *ver1* (*aflM*) significantly decreased on day 16 compared to day 12 but returned to near-initial levels by day 21 (Figure 1). In contrast, *ordA* (*aflQ*) expression significantly and progressively increased on days 16 and 21 (Figure 1E).

The expression of the *omtA* gene significantly declined on day 16 compared to day 12. However, on day 21, it rose again to a fold change (FC) close to the initial level (Figure 1B), showing a similar tendency to *aflR* mRNA expression.

The expression of the *cypA* gene significantly declined on days 16 and 21 compared to that on day 12. The samples collected on days 21 and 16 did not differ significantly (Figure 1C).

The expression of the *ver-1* gene significantly declined on day 16 compared to day 12. However, on day 21 it showed a partial recovery but did not fully return to the day 12 level (Figure 1D). There was no statistically significant difference between day 16 and day 21.

The mRNA expression levels in the 16- and 21-day samples did not differ significantly.

The expression of the *aflQ* (*ordA*) gene at the mRNA level showed a significant increase at both 16 and 21 days. The 21-day value also showed a significantly higher expression at the mRNA level compared to day 16, but it did not reach statistical significance (*p* = 0.109) (Figure 1E).

### 2.2. mRNA Expression Analysis of A. flavus Zt41 Genes Responsible for Toxin Production at 30 °C and the Effect of L. fermentum Co-Inoculation

**Figure 2:**
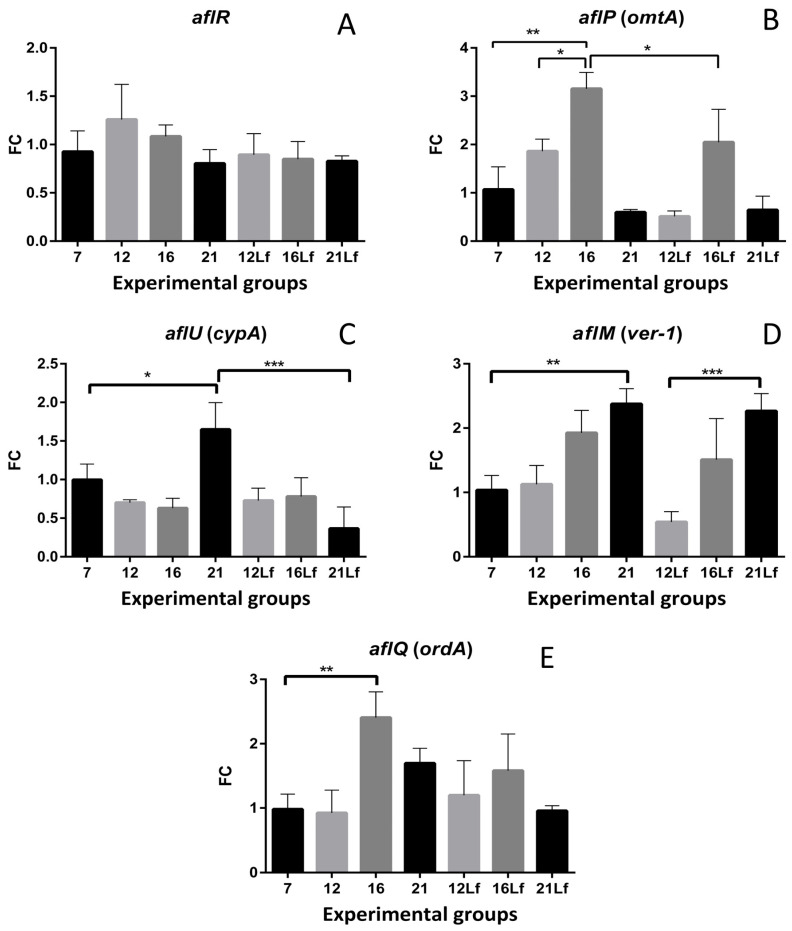
mRNA expression of *aflR* (**A**)*, aflP* (*omtA*) (**B**), *aflU* (*cypA*) (**C**), *aflM* (*ver-1*) (**D**), and *aflQ* (*ordA*) (**E**) genes from total RNA of *A. flavus* samples incubated at 30 °C on days 7, 12, 16, and 21. FC—Fold Change. Days 7, 12, 16, and 21 were used for sampling. Lf denotes samples supplemented with *L. fermentum*. The deviations were plotted with SD (Standard Deviation) values. The significances were *p* < 0.05 *, *p* < 0.01 **, and *p* < 0.001 ***.

At 30 °C, *aflR* expression remained stable over time and was unaffected by *L. fermentum* co-inoculation (Figure 2A). In the absence of *L. fermentum*, transient peaks in *omtA*, *cypA*, *ver1*, and *ordA* expression were observed between days 12 and 16. Co-inoculation with *L. fermentum* significantly suppressed the expression of *omtA* (days 12 and 16), *cypA* (day 21), and *ordA* (day 16) compared to the respective controls (Figure 2).

The expression of the *omtA* (*aflP)* gene at the mRNA level during incubation at 30 °C on days 12 and 16 showed an increase compared to the 7-day sample (Figure 2), but only the increase in *omtA* expression at the mRNA level in the 16th day sample was statistically significant (*p* = 0.004). The 12- and 16-day samples also differed significantly from each other (*p* = 0.0103). By day 21, *omtA* expression did not differ from that on day 7. Supplementation with *L. fermentum* significantly reduced the value compared to that of the non-bacterial sample on day 12 (*p* = 0.0073). At day 16, the highest expression levels were observed, and the addition of *L. fermentum* to the culture significantly reduced *omtA* mRNA expression compared to the parallel 16-day sample (*p* = 0.0318) (Figure 2B).

The expression of the *cypA* (*aflU*) gene at the mRNA level during incubation at 30 °C on days 12 and 16 did not change. In contrast, there was a significant increase in mRNA expression at day 21 compared to that at day 7 (Figure 2) (*p* = 0.033). The supplementation of *L. fermentum* resulted in a significant reduction in *cypA* expression compared to that in the non-bacterial sample at day 21 (*p* = 0.0003) (Figure 2C). There was no significant difference between the *L. fermentum*-supplemented and untreated samples at day 12 and 16 in mRNA-level *cypA* expression.

The expression of *ver-1* (*aflM*) at the mRNA level during incubation at 30 °C on days 12 and 16 did not differ significantly, despite the apparent increase on day 16. In contrast, there was a significant increase in mRNA expression at day 21 compared to that at day 7 (*p* = 0.0084) (Figure 2). The addition of *L. fermentum* did not effectively reduce the expression of *ver-1* at the mRNA level on either day 16 or 21. A significant upward trend was observed compared to samples day 7, day 12, and day 12Lf (*p* = 0.0004) (Figure 2D). The rate of increase was almost the same as that of the untreated samples.

The expression of the *aflQ* (*ordA*) gene at the mRNA level during incubation at 30 °C did not differ significantly in the day 12 samples. Significantly increased expression on day 16 (*p* = 0.0024) was similar to that observed on day 21, which, however, showed a slight decrease compared to day 16, but did not show statistically significant differences in mRNA expression levels compared to day 7 (Figure 2E). These results indicate a clear suppressive effect of the bacterium on *ordA*, a key late-pathway gene in aflatoxin biosynthesis.

### 2.3. mRNA Expression Analysis of A. flavus Zt41 Genes Responsible for Toxin Production at 37 °C and the Effect of L. fermentum Co-Inoculation

**Figure 3:**
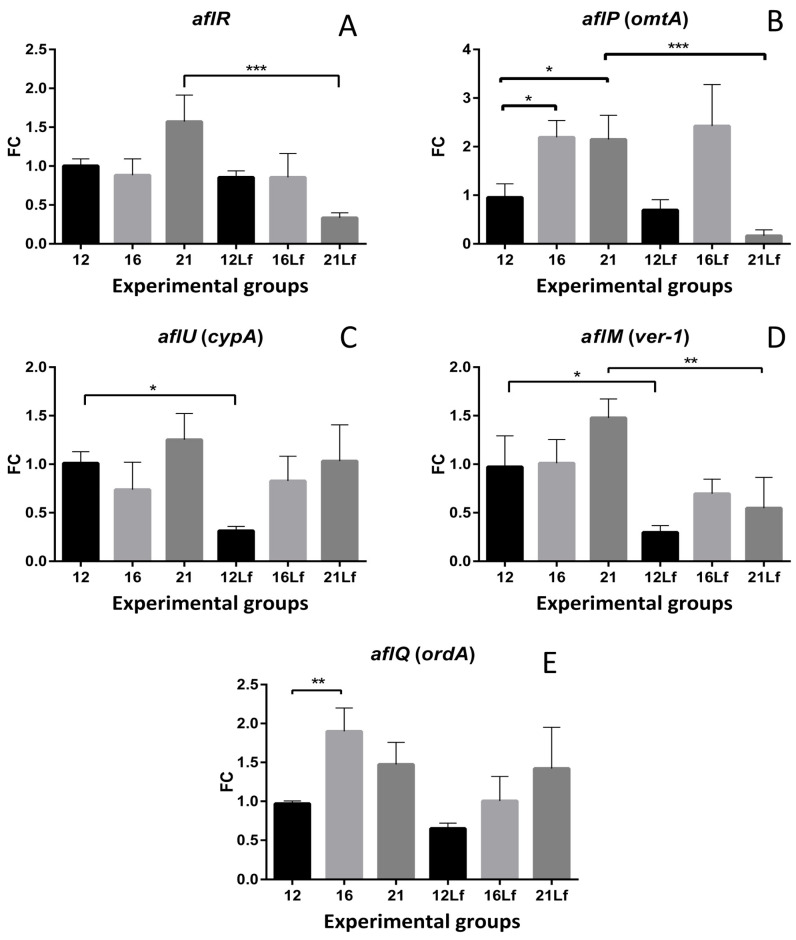
*aflR* (**A**)*, aflP* (*omtA*) (**B**), *aflU* (*cypA*) (**C**), *aflM* (*ver-1*) (**D**), and *aflQ* (*ordA*) (**E**) genes from total RNA of *A. flavus* samples incubated at 37 °C on days 12, 16, and 21. FC—Fold Change. Sampling days are 12, 16, and 21. Lf denotes samples supplemented with *L. fermentum*. The deviations were plotted with SD (Standard Deviation) values. The significances were *p* < 0.05 *, *p* < 0.01 **, and *p* < 0.001 ***. Note: The day-7 baseline (pre-*L. fermentum* inoculation) is presented only in Figure 2 (30 °C), as this time point served as the reference before bacterial addition. For the 37 °C experiment, sampling began at day 12.

At 37 °C, *aflR* expression showed a non-significant increase by day 21. Samples co-inoculated with *L. fermentum* (21Lf) exhibited significantly lower *aflR* expression than the untreated day-21 samples (*p* = 0.001) (Figure 3A).

The expression of *omtA* (*aflP*) was elevated on days 12 and 16 compared to day 12 in the untreated samples. *L. fermentum* supplementation significantly reduced expression on day 21 (*p* = 0.0007). On day 16, *omtA* expression remained significantly higher than on day 12 even in the presence of *L. fermentum* (*p* = 0.0041 and 0.0026 vs. day 12 and 12Lf, respectively) (Figure 3B).

The expression of *cypA* (*aflU*) did not show significant elevation on days 16 and 21 compared to day 12. *L. fermentum* significantly reduced *cypA* expression on day 12 (*p* = 0.0224). On day 21, expression in the *L. fermentum* group was higher than on day 12Lf (*p* = 0.0283) but not higher than the untreated day-12 level (Figure 3C).

The expression of *ver1* (*aflM*) remained stable on days 16 and 21 compared to day 12. *L. fermentum* supplementation significantly lowered expression on day 12 (*p* = 0.0476) and on day 21 (*p* = 0.006) compared to the respective untreated controls (Figure 3D).

The expression of *ordA* (*aflQ*) was significantly elevated on days 16 and 21 compared to day 12 (*p* = 0.0043). Although *L. fermentum* co-inoculation did not produce statistically significant differences, a clear trend toward reduced expression was observed (Figure 3E).

### 2.4. Aflatoxin B1 Production of A. flavus Zt41 Under the Studied Conditions

**Figure 4:**
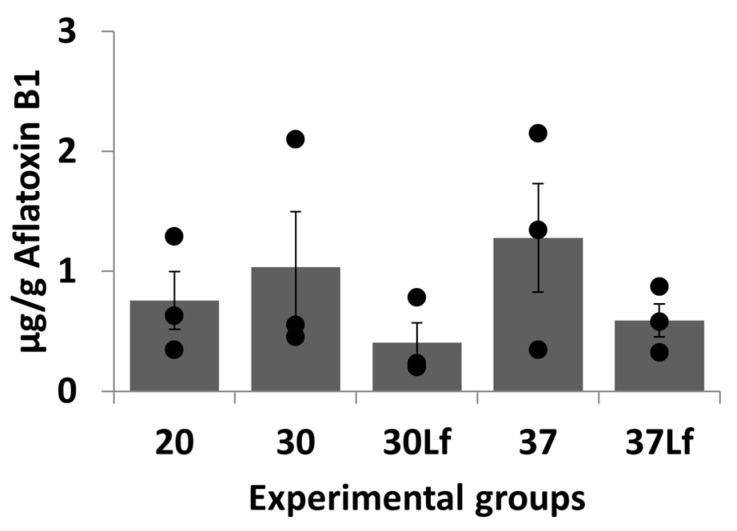
Aflatoxin B1 production by *A. flavus* Zt41 at three temperatures (20 °C, 30 °C, and 37 °C). Lf denotes samples supplemented with *L. fermentum*. The samples were collected after 21 days of incubation. Error bars represent standard deviation (SD, n = 3). A consistent reduction in aflatoxin production (two-way ANOVA, *p* = 0.132) was observed in the presence of *L. fermentum*.

Aflatoxin B1 production by *A. flavus* Zt41 in corn silage at the end of the experiment (day 21) at three different temperatures (20 °C, 30 °C, and 37 °C) with and without co-inoculation with *L. fermentum* is shown in Figure 4. An increasing trend in AFB1 concentration was observed with rising temperature, although the differences did not reach statistical significance due to high inter-replicate variability. Co-inoculation with *L. fermentum* produced a consistent numerical reduction in AFB1 levels at all three temperatures, with decreases of approximately 50–60% (two-way ANOVA, *p* = 0.132). Individual data points are presented in Figure 4 to illustrate the variability.

## 3. Discussion

*A. flavus* is a saprophytic fungus with a broad temperature growth range, with an optimum temperature for aflatoxin B1 (AFB1) production classically reported to be 28–30 °C [27]. However, recent studies under climate change scenarios have shown that certain strains can maintain or increase toxin synthesis at temperatures up to 37 °C, particularly when combined with water stress or fluctuating conditions typically encountered during ensiling [8,19,28]. In the present study, AFB1 concentrations showed a tendency to increase with rising temperature (Figure 4), although inter-replicate variability prevented the differences from reaching statistical significance. Nevertheless, the transcript levels of the regulatory gene *aflR* and most structural genes were higher at 37 °C than at 20 °C, indicating that elevated temperature can activate the aflatoxin biosynthetic cluster, even in a complex silage matrix [18,20,21,29]. The pathway-specific regulator AflR exhibited increased expression at 37 °C (Figure 3), consistent with observations in maize kernels [19], pistachio nuts [13], and controlled in vitro systems [30]. Temperature is a key environmental factor influencing aflatoxin biosynthesis in *A. flavus*, with increased expression of biosynthetic genes typically observed between 30 and 37 °C. This regulation is mediated, at least in part, by global regulatory systems such as the velvet complex (VeA–VelB–LaeA), which integrates environmental cues into secondary metabolism [31]. The transient peaks in *omtA* (*aflP*), *cypA* (*aflU*), *ver1* (*aflM*), and *ordA* (*aflQ*) expression observed at 30 °C between days 12 and 16 (Figure 2), followed by a decline on day 21, suggest a temperature-dependent “pulse” of biosynthetic activity. Co-inoculation with *L. fermentum* counteracted temperature-induced activation of the aflatoxin cluster. At all tested temperatures, *L. fermentum* produced a consistent numerical reduction in the final AFB1 concentration (Figure 4), corroborating previous reports on the efficacy of lactic acid bacteria in inhibiting *A. flavus* growth and aflatoxin production in cereal grains and kernels, including maize [32]. Although this reduction did not reach statistical significance (two-way ANOVA, *p* = 0.132), the magnitude of the decrease (approximately 50–60%) indicates a biologically meaningful inhibitory effect.

The magnitude of the observed numerical reduction (≈50–60%) is biologically meaningful and comparable to a recent study in which lactic acid bacteria inoculation lowered AFB1 by approximately 40% in *A. flavus*-contaminated experimental corn silage after two months of storage [33]. Our qRT-PCR data extend these phenotypic observations by demonstrating that the inhibitory effect is, at least partly, mediated by strong transcriptional repression of *aflR* and the entire biosynthetic cluster, including the late-pathway gene *ordA*—a mechanism previously shown for *Lactiplantibacillus plantarum* [26] but only rarely documented for *L. fermentum*. Complementary in vitro work has confirmed the potent antifungal activity of several *L. fermentum* strains against aflatoxigenic *A. flavus* isolates [34], further supporting the strain-specific biocontrol potential observed in the present silage model system.

More importantly, the qRT-PCR results provide molecular evidence that the observed numerical reduction in AFB1 is associated with transcriptional repression of several key genes in the aflatoxin biosynthetic pathway. Co-inoculation with *L. fermentum* led to significant downregulation of *aflR* and multiple structural genes (omtA, cypA, and *ver1*) at both 30 °C and 37 °C, although the timing, magnitude, and consistency of the effect varied between genes and temperatures (Figure 2 and Figure 3). A particularly pronounced suppression was observed for the late-pathway gene ordA at 30 °C, while at 37 °C the inhibitory effect was most evident on the regulatory gene *aflR* and *omtA*. These differential responses indicate that temperature may influence the sensitivity of individual genes or steps within the aflatoxin biosynthetic pathway to the inhibitory action of *L. fermentum*, consistent with previous observations that the aflatoxin cluster shows temperature-dependent regulation at both transcriptional and post-transcriptional levels [18,20,28]. The stronger suppression of *ordA*, which encodes a cytochrome P450 monooxygenase catalyzing a rate-limiting final step, at 30 °C may be particularly important for reducing toxin accumulation. Several putative mechanisms may contribute to this gene-specific repression. First, organic acids (lactic, acetic, and phenyllactic acids) produced by *L. fermentum* lower the local pH, creating an acidic environment that impairs AflR binding to promoter regions [35,36]. Second, LAB-derived hydrogen peroxide and reactive oxygen species may induce oxidative stress responses in *A. flavus*, which can modulate aflatoxin biosynthesis through global regulatory pathways involving the velvet complex (VeA, VelB, and LaeA) [37]. Third, the cell wall components, exopolysaccharides, and biosurfactants of *L. fermentum* can adhere to fungal hyphae, potentially affecting fungal growth and aflatoxin production through physical interactions, competitive inhibition, and toxin binding mechanisms [38]. A particularly interesting observation was the pronounced suppression of the late-pathway gene *ordA* at 30 °C in the presence of *L. fermentum* (Figure 2). The *ordA*-encoded cytochrome P450 monooxygenase catalyzes the conversion of O-methylsterigmatocystin (OMST) to AFB1 and is considered a rate-limiting enzyme in the late step [39]. Similar transcriptional inhibition of aflatoxin biosynthetic genes has been reported for plant phenolics and essential oils [14], but is still rarely documented for lactic acid bacteria, making this a valuable insight for designing more effective multi-hurdle biocontrol strategies with LAB.

From an applied perspective, the robust inhibitory effect observed at 37 °C is particularly encouraging. Silage fermentation frequently experiences elevated temperatures, particularly in tropical/subtropical regions or during hot summers in temperate zones [40,41]. The ability of *L. fermentum* to suppress both gene expression and toxin production under such conditions supports its potential as a targeted silage inoculant for aflatoxin control, either alone or in combination with established homofermentative strains that primarily improve lactic fermentation and aerobic stability.

It should be noted that the experimental system used sterilized corn silage supplemented with distilled water. While this design allowed controlled investigation of temperature and *L. fermentum* effects in a post-harvest matrix, it did not fully replicate the complex anaerobic fermentation dynamics, pH evolution, or indigenous microbial community of real ensiling conditions. Therefore, the present findings primarily demonstrate direct effects on *A. flavus* gene expression and toxin production. Future studies using non-sterilized silage under realistic ensiling conditions will be necessary to validate these results in situ.

In conclusion, temperature is a powerful environmental trigger of the aflatoxin biosynthetic cluster in *A. flavus* colonizing corn silage, with maximal induction observed at 37 °C. *L. fermentum* effectively counteracts this induction through mechanisms that include significant downregulation of *aflR* and key structural genes, resulting in a numerically consistent reduction in AFB1 accumulation. These findings support the incorporation of selected *L. fermentum* strains into silage inoculant formulations, particularly in regions prone to high temperatures during ensiling.

## 4. Conclusions

This study demonstrates that temperature is a major driver of aflatoxin biosynthetic gene expression in *A. flavus* colonizing corn silage, with the highest transcript levels of *aflR* and structural genes observed at 37 °C. Co-inoculation with *L. fermentum* effectively counteracted this temperature-induced activation by significantly down-regulating the expression of key pathway genes (*aflR*, *omtA*, *cypA*, *ver1*, and *ordA*) at 30 °C and 37 °C. At all tested temperatures, *L. fermentum* produced a consistent numerical reduction in the final AFB1 concentration (approximately 50–60%), although this decrease did not reach statistical significance (two-way ANOVA, *p* = 0.132). The strongest suppressive effects on both gene expression and toxin accumulation were observed at 30 °C and 37 °C, temperatures that are increasingly relevant under current and projected climate-change scenarios.

These findings provide clear molecular (qRT-PCR) evidence that *L. fermentum* acts, at least in part, through strong transcriptional repression of the entire aflatoxin biosynthetic cluster, including the late-pathway gene *ordA*. The results support the incorporation of selected *L. fermentum* strains into silage inoculant formulations as a practical biocontrol strategy to mitigate AFB1 contamination in maize silage, particularly in regions prone to elevated ensiling temperatures. Further field-scale trials under realistic ensiling conditions are warranted to validate these laboratory observations and to optimize application protocols for commercial silage production.

## 5. Materials and Methods

### 5.1. Microorganisms

The *Aspergillus flavus* Zt41 (NCAIM F.01021) strain with good aflatoxin B1 production capacity was cultivated on potato dextrose agar (PDA; VWR) for 7 days at room temperature. Hyphae and spores were collected from the plate surfaces using sterile distilled water. The suspension was homogenized with a Potter–Elvehjem device, spore numbers were counted with a Bürker chamber under a microscope (KERN OBN 147 fluorescent microscope, Badlingen, Germany), and the concentration of the suspension was set to 10^8^ spores/mL for use as an inoculum in the experiments.

*Limosilactobacillus fermentum* (NCAIM B.02688) strain with a good ability to hinder the growth of *Aspergillus flavus* Zt41 (preliminary studies, results not shown here) was cultivated in MRS broth (de Man, Rogosa & Sharpe medium; VWR) for 2 days at 37 °C. Cell numbers were enumerated using a Bürker chamber under a microscope (KERN OBN 147 fluorescent microscope, Badlingen, Germany), and the concentration of the suspension was set to 10^8^ cells/mL to be used as the inoculum in the experiments.

### 5.2. Experimental Setup

Eighteen 50 mL capped bottles were filled with 10 g of corn silage and 6 mL of distilled water. The bottles were left to stand for 1 h and then sterilized. The bottles were inoculated with 200 µL of *A. flavus* Zt41 suspension and incubated for 7 days at room temperature in the dark. Three bottles were used as 7-day controls.

After one week of incubation with *A. flavus*, *L. fermentum* was added to bottles designated for the 30 °C and 37 °C conditions **. All bottles were then incubated at their respective temperatures (20 °C, 30 °C, or 37 °C) for two additional weeks.

The bottles were incubated at three different temperatures (20, 30, and 37 °C) for two weeks in the dark. The experimental setup and sample coding are described in Table 1.

### 5.3. Sampling for Toxicological Studies

To determine aflatoxin B1 contents, 1 g was collected on the 21st day (the end) of the experiment from all 15 bottles into sterile plastic tubes. To each sample, 2 mL of methanol was added, and the tubes were vortexed vigorously for 1 min. The samples were then left in the dark at room temperature for 24 h. After incubation, the samples were mixed again for 1 min and centrifuged at 3000 rpm for 10 min at 20 °C. The supernatants were transferred to clean tubes and stored at −20 °C until analysis.

### 5.4. Aflatoxin B1 Measurement by HPLC Method

For AFB1 measurement, 1 mL of each extract was evaporated and resuspended in 0.4 mL hexane, followed by the addition of 0.1 mL trifluoroacetic acid (TFA) and derivatization at 60 °C for 15 min. Then, 0.4 mL of water:acetonitrile (9:1) was added to the mixture. After mixing, the lower phase was collected and 3 µL was injected onto a Prodigy C18 150 × 4.6 mm 5 µm column (Phenomenex, Torrance, CA, USA) in a modular Shimadzu HPLC system equipped with an RF-20A fluorescence detector (Shimadzu, Duisburg, Germany). The separation was carried out at a flow rate of 1 mL/min using isocratic elution (65:35) of water and a mixture of methanol:acetonitrile (1:1, *v*/*v*). The detector wavelengths were 350 and 430 nm for excitation and emission, respectively.

### 5.5. Sampling for Expression Studies Using qRT-PCR

Samples were taken from three parallel silages (from which samples were also taken for toxin analysis) after the first week of incubation, and then the different settings were sampled on days 12, 16, and 21. For RNA sampling, sterile platinum inoculation loops were used to acquire 6–8 loopfuls of biomass by scraping the surface of the silage, which were placed in a nuclease-free sterile centrifuge tube and stored at −80 °C until further processing. The samples were stored at −80 °C and processed on dry ice or ice in every case.

### 5.6. Isolation of Total RNA Using Geneaid Total RNA Mini Kit

To a sterile centrifuge tube containing conidia and mycelia, 400 µL of Tri Reagent (Zymo Research, Irvine, CA, USA) was added, and the sample was thoroughly mixed. The sample mixture was transferred to 1.5 mL microtubes with screw caps, into which sterile, 3 mm diameter stainless steel beads were added. Cell disruption and homogenization were performed using a Minilys homogenizer (Bertin Technologies, Montigny-le-Bretonneux, Montigny-le-Bretonneux, France) at 4000 rpm for 20 s. The homogenization cycle was repeated once for each sample, followed by incubation on ice for 5 min. After cell disruption, the samples were centrifuged (14,000× *g*, 10 min, 4 °C) and the supernatant was transferred into a new sterile centrifuge tube, then 400 µL of chloroform (equal volume) was added. The sample was mixed (IKA MS2 Minishaker, Staufen im Breisgau, Germany) and centrifuged (14,000× *g*, 10 min, 4 °C), and the supernatant in the upper phase was transferred to new sterile tubes.

Next, the protocol provided by the manufacturer of the Geneaid Total RNA Mini Kit was followed. To the supernatant, 400 µL of RB buffer was added, mixed, and 400 µL of 96% ethanol was added, which was mixed immediately with the sample by repeatedly turning over the tube to avoid nucleic acid precipitation. Then, 700 μL was pipetted into tubes containing RNA binding membrane provided by the manufacturer and centrifuged at 14,000× *g* for 1 min at 4 °C. The flow-through was discarded, and the remaining sample was pipetted onto a membrane. The sample was centrifuged according to the previous parameters, and the flow was discarded.

Next, the membrane on which the RNA was bound was washed in several steps. First, 400 μL of W1 buffer was added, centrifuged (14,000× *g*, 30 s, 4 °C), and the flow-through was discarded. In the second washing process, the same washing steps were repeated twice by pipetting 600 µL of wash buffer onto the samples, centrifuging (14,000× *g*, 30 s, 4 °C), and discarding the flow-through solution. Then, in the membrane drying step prior to RNA elution, the microtubes were centrifuged at 14,000× *g* for 3 min at 4 °C. Next, 50 µL of nuclease-free water was added to the center of the membrane, and after 2 min, the membrane-bound RNA was eluted by centrifugation (14,000× *g*, 1 min, 4 °C), and the sample was immediately placed on ice. The concentration and purity of the samples were determined using a NanoDrop 2000 spectrophotometer (Thermo Fisher Scientific, Waltham, MA, USA). The samples were stored at −20 °C).

### 5.7. Preparation of cDNA

Samples were arranged in groups according to the incubation temperature used in the experiment (20 °C, 30 °C, and 37 °C), and cDNA was prepared from these groups. From the same concentration of diluted RNA samples, the reaction mixtures were prepared using the components of the High-Capacity cDNA Reverse Transcription Kit (Applied Biosystems, Thermo Fisher Scientific, Vilnius, Lithuania.) as follows: amounts of components (μL) in 10 μL reaction volume: 10x RT buffer 2; 10x Random primers 2; 25x dNTP mix (100 mM) 0.8; Multiscribe TM reverse transcriptase 0.5. The volume was brought up to 10 μL with nuclease-free water.

RNA samples with the set concentration were transferred to sterile PCR tubes, and 10 μL of the assembled MasterMix was added to each sample. The mixtures were then mixed and centrifuged. The time and temperature profile of the cDNA preparation was as follows: step 1—25 °C for 10 min; step 2—37 °C for 120 min; step 3—85 °C for 5 min; and step 4—4 °C.

### 5.8. Designing Primers for Toxin Production Genes (aflR, cypA, ordA, omtA, ver1)

The primers required for qPCR, approximately 18–25 nucleotide long oligonucleotides, were designed using Primer-Blast and Primer Express 3.0 programs to complement the coding region of the gene of interest, in this case, the genes regulating the production of toxins. In addition to the specific primers, we also designed primers for the so-called housekeeping genes (*cox5* and *hisH4*, which are the reference genes in the qPCR reaction), which are constantly expressed in *A. flavus* [39,42]. Oligonucleotides were synthesized by Eurofins Genomics. The following table includes the primers designed for genes involved in the production of *A. flavus* toxin and used in qPCR (Table 2).

After cDNA synthesis, the samples were diluted tenfold with nuclease-free water. The primers were diluted to the same concentration with nuclease-free water to give a uniform concentration of 5 pmol/µL in the reaction mixture during the qPCR reaction. Individual primer pairs (“forward” and “reverse” primers) and a green, fluorescent nucleic acid dye (EvaGreen, Biotium, San Jose, CA, USA) were added to the pre-assembled MasterMix (Light Cycler 480 Probes). The components (μL) for the qRT-PCR reaction mixture were as follows: MasterMix 5; primer pair 1. The mixture was brought to 8 μL with nuclease-free water. First, 2 µL of diluted cDNA samples were pipetted into 96-well reaction plates (Applied Biosystems MicroAmp Fast 96-well reaction plate, Suzhou, China), and 8 µL of the MasterMix containing primers and EvaGreen dye were added to each well. Two replicates were prepared for each sample using gene-specific primers and primers for housekeeping genes. The sample-filled PCR plate was sealed with a transparent lid (AB MicroAmp Optical Cap Strip, Suzhou, China).

The temperature and time profile of the qRT-PCR was adjusted using the parameters listed in Table 3 for each primer.

qPCR was performed using a StepOnePlus Real-Time PCR System (Applied Biosystems, Singapore, Singapore). The reaction parameters were set, and the results were evaluated using StepOne v2.3 software. The expression of genes responsible for toxin production was quantified using the comparative Ct (2^−ΔΔCt^) method.

### 5.9. Determining the Efficiency of Primers

The efficiency of the primers was determined by generating a standard curve using a 1:5 dilution series of the PCR products. The results are presented in Table 4.

### 5.10. Statistical Evaluation

Statistical analyses were performed using GraphPad Prism (version 6.0). Normality tests were performed using the Kolmogorov–Smirnov test. Samples with a normal distribution were analyzed using one-way analysis of variance (ANOVA) and Bonferroni post hoc tests. The significance level was set at *p* < 0.05. The deviations are plotted with standard deviation (SD) values.

## Figures and Tables

**Table 1:** Experimental setup and the coding of samples.

	20 °C	30 °C	37 °C
without *Limosilactobacillus fermentum*	20	30	37
with *Limosilactobacillus fermentum*		30-Lf	37-Lf

**Table 2:** Primers designed for genes involved in the toxin production of *Aspergillus flavus*.

Primer	Sequence	GC	Tm
*aflR*f	CAGGTCGGAACAGGGACTTC	60	61
*aflR*r	AGAGCGTGTGGTGGTTGATT	50	57
*ver1*f	TTGTTCGCTGCATGGCAATC	50	57
*ver1*r	TTACCCATTCGGCTGCATCA	50	57
*omtA*f	GTCACGGCAGATGAGACGAA	55	59
*omtA*r	GGAACCGGGCCATGAACTAA	55	59
*ordA*f	GAAAAGACTCCCCGCCAGAA	55	59
*ordA*r	GCCCAAAGCCGAACACAAAT	50	57
*cypA*f	CTTGGCCGGTGTTGTCAATG	55	59
*cypA*r	ATCGACGGGTCCATGCATAC	55	59
**Reference gene primer**			
*cox5*f	CGTCATTCACTTGTTCGCTAAG	45	58
*cox5*r	CCTTGGCATACTCGTTGGAAG	52	59
*hish4*f	TCGTCGTGGTGGTGTCAAG	57	58
*hish4*r	TTGGCGTGCTCAGTGTAGG	57	58

**Table 3:** Temperature and time profile of qPCR.

qPCR Cycle	Denaturation	Denaturation (40 Cycles)	Amplification (40 Cycles)
	Step 1	Step 2	Step 3
Temperature	95 °C	95 °C	60 °C
Time	10 min	0.25 min	0.5 min

**Table 4:** Efficiency of primers.

Primers	R^2^	Efficiency (%)
*cox5*	0.995	94.47
*hisH4*	0.986	93.8
*aflR*	0.976	91.56
*omtA*	0.967	86.76
*cypA*	0.976	88.96
*ver-1*	0.992	86.69
*ordA*	0.972	92.36

## Data Availability

The authors declare that the data supporting the findings of the study are available in the study. If raw data files are required, they are available on reasonable request from the corresponding author.
